# Pharmacological inhibition of toll-like receptor 4 suppresses ischemia-reperfusion injury-induced inflammation and improves lung allograft function after transplantation

**DOI:** 10.1016/j.jhlto.2025.100399

**Published:** 2025-09-26

**Authors:** Yasufumi Goda, Mamoru Takahashi, Itsuki Yuasa, Yumeta Shimazu, Naoki Date, Satona Tanaka, Hiroyuki Katsuragawa, Akihiro Ohsumi, Daisuke Nakajima, Hiroshi Date

**Affiliations:** a Department of Thoracic Surgery, Graduate School of Medicine, Kyoto University, Kyoto, Japan; b Department of Diagnostic Pathology, Graduate School of Medicine, Kyoto University, Kyoto, Japan

**Keywords:** Toll-like receptor 4, Ischemia reperfusion injury, Inflammation, Lung transplantation, Macrophage

## Abstract

Ischemia reperfusion injury (IRI) is a significant risk factor for primary graft dysfunction following lung transplantation. Toll-like receptor 4 (TLR4) signaling plays an important role not only in IRI but also in the development of acute and chronic allograft rejection. We investigated the therapeutic effect of Viral Inhibitory Peptide for TLR4 (VIPER), a pharmacological TLR4-inhibitory peptide, in a clinically relevant murine lung transplantation model. VIPER-treated lungs demonstrated improved function, with reduced mean airway pressure and increased compliance and inspiratory volume. The wet-to-dry weight ratio was significantly reduced, indicating decreased pulmonary edema. Inflammatory cytokines monocyte chemoattractant protein-1 (MCP-1), interferon-gamma (IFN-γ), and interleukin-6 (IL-6) were significantly decreased, with a trend toward lower levels of the fibrogenetic cytokine transforming growth factor-beta (TGF-β). Histological evaluation revealed reduced acute lung injury scores and fewer inducible nitric oxide synthase (iNOS)-positive inflammatory cells. These findings demonstrate that pharmacological inhibition of TLR4 with VIPER effectively attenuates IRI-associated inflammation and improves early graft function. Such pharmacological TLR4-targeting strategies might represent a therapeutic approach in lung transplantation.

Ischemia-reperfusion injury (IRI) is an inevitable complication of deceased-donor lung transplantation and a major contributor to primary graft dysfunction, the leading cause of early post-transplant mortality.^1^ The pathogenesis of IRI is driven by the release of damage-associated molecular patterns from injured cells, which initiate an inflammatory cascade primarily through Toll-like receptor 4 (TLR4) signaling.^2^ This activation leads to the recruitment of immune cells, including neutrophils, monocytes, and inflammatory macrophages, ultimately resulting in increased vascular permeability and lung injury.

In preclinical models, recent studies from the Washington group using hilar clamping and transplant models demonstrated that lung-infiltrating B cells produce the monocyte chemokine CCL7 in a TLR4–Toll/interleukin-1 receptor(TIR)-domain-containing adapter-inducing interferon-β–(TRIF) dependent manner. This mechanism promotes classical monocyte recruitment and subsequent neutrophil extravasation, exacerbating lung dysfunction.^3^ These findings highlight the critical role of TLR4-mediated signaling in the pathogenesis of lung IRI in animal models.

The viral inhibitory peptide for TLR4 (VIPER) is an 11-amino-acid peptide derived from the vaccinia virus A46 protein. VIPER selectively inhibits TLR4 signaling by binding to adaptor proteins, including MyD88 adaptor-like (Mal) and TRIF-related adaptor molecule, thereby blocking downstream mitogen-activated protein kinase activation and transcription factor signaling.^4^

In this study, we investigated the effect of TLR4 inhibition using VIPER in a murine lung transplantation model, with particular focus on the inflammatory profile of the lung allograft. To the best of our knowledge, this is the first study to examine the therapeutic impact of pharmacological TLR4 inhibition in a clinically relevant murine lung transplantation model, rather than in conventional hilar clamp-based models.

Both donor and recipient mice (C57BL/6J) were obtained from Charles River Laboratories, Japan Inc. (Kanagawa, Japan). All mice were male, aged 8-12 weeks, and weighed between 24 and 30 g. The animals were housed in a specific pathogen-free facility at Kyoto University, Japan, and were cared for in accordance with institutional guidelines. The experimental protocol was approved by the Institutional Animal Care Committee (Approval Number: MedKyo22569). The heart-lung block was harvested from donor C57BL/6J mice after flushing with ET Kyoto solution. Subsequently, the heart-lung block was immersed in ET Kyoto solution and preserved at 4 °C for 18 hours. Thirty minutes before lung transplantation, VIPER®, a TLR4 inhibitory peptide, or CP7, a control peptide (both from Novus Biologicals, Centennial, CO, USA), was administered via the penile vein (1.0 mg/kg in 0.5 mL PBS solution).^5^ Orthotopic left lung transplantation was performed on C57BL/6J recipient mice following previously established protocols.^6^ After 2 hours of reperfusion, the mice were sacrificed, and the lung allografts were collected for evaluation (Figure 1A). Lung function was assessed using a rodent ventilator (FlexiVent, SCIREQ, Montreal, QC, Canada). During the evaluation, the right pulmonary hilum, including the accessory lobe, was secured with a vascular clamp (Figure 1B). Parameters such as mean airway pressure, compliance, and inspiratory capacity were measured as previously described. Following pulmonary function testing, the left lung was harvested and divided into two parts. The lower portion was used to determine the lung wet-to-dry weight (W/D) ratio. Wet weight was measured immediately after harvesting, and dry weight was measured after 24 hours of desiccation at 100 °C. The W/D ratio was calculated by dividing the wet weight by the dry weight. To confirm the reproducibility of our findings, we separately performed an additional set of lung transplantation to obtain tissue for cytokine quantification and histological analysis. Lung tissue (25 mg) was excised and homogenized in 800 μL of buffer containing protease and phosphatase inhibitors. The homogenate was centrifuged at 10,000 × *g* for 20 min, and the supernatant was collected for cytokine analysis using enzyme-linked immunosorbent assay (ELISA). Sandwich ELISA kits (Abcam, Cambridge, UK) were used to quantify interleukin-6 (IL-6), interferon-gamma (IFN-γ), transforming growth factor-β（TGF-β), and monocyte chemoattractant protein-1 (MCP-1). Lung allografts were longitudinally sectioned, fixed in 10% formalin, embedded in paraffin, and sectioned at a thickness of 5 µm. Sections were stained with hematoxylin and eosin (H&E) for histological evaluation. Immunohistochemistry was performed on deparaffinized and rehydrated tissue sections (5 µm thick) using standard protocols. The following primary antibodies were utilized: rabbit anti-CD206 (1:400; Cell Signaling Technology, Danvers, MA) and rabbit anti-inducible nitric oxide synthase (iNOS) (1:100; Abcam, Cambridge, UK). Histological scores of acute lung injury were graded on a scale based on appearance: normal (0%), mild (<10%), moderate (10%-50%), or severe (>50%) abnormalities, and were scored as 0, 1, 2, and 3, respectively.^7^ Ten randomly selected alveolar areas at high-power fields from each lung section were analyzed, and the number of CD206- and iNOS-positive cells was quantified using ImageJ2 software (NIH, Bethesda, MD, USA). The mean value from these ten areas was calculated for each sample.

**Figure 1:**
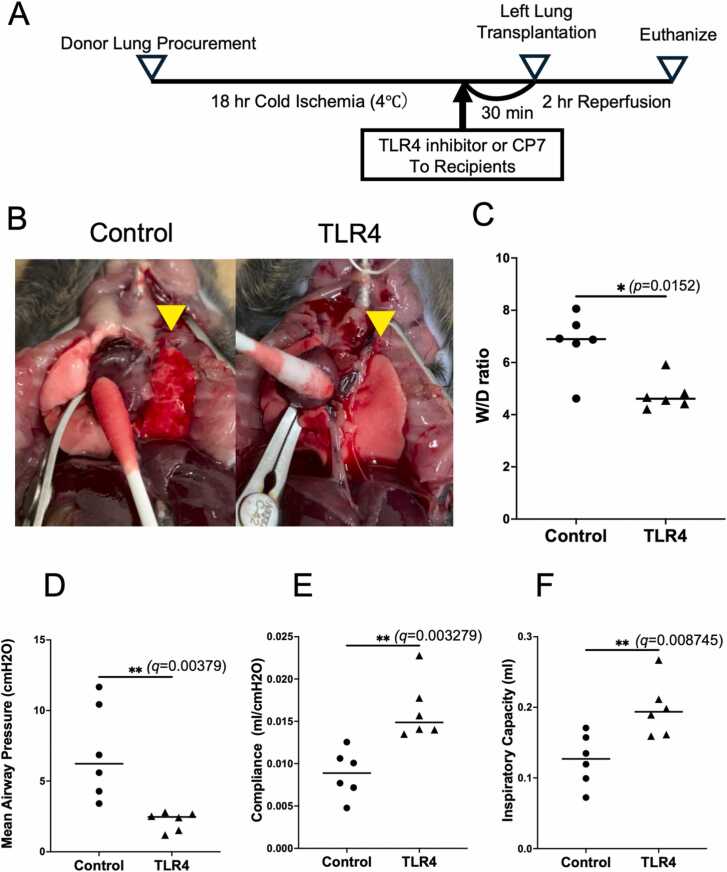
TLR4 inhibition significantly ameliorates lung injury and improves graft function, as demonstrated by macroscopic findings. **(A)** Schematic of the experimental design. **(B)** Representative gross images of the transplanted left lung at 2 hours after reperfusion (yellow arrowheads indicate the transplanted lungs). **(C)** Quantification of pulmonary edema using the wet-to-dry weight ratio (*n* = 6). **(D-F)** Lung function parameters, including Mean Airway Pressure **(D)**, Compliance **(E)**, and Inspiratory Capacity **(F)**, are shown. Sample size: *n* = 6 for each group. *P < 0.05. **q < 0.01. Bar indicates the mean value.

Statistical analyses were performed using GraphPad Prism 9 (GraphPad Software, Boston, MA, USA). *P* values < 0.05 were considered statistically significant, as determined by the Mann-Whitney *U* test. To control for the false discovery rate due to multiple comparisons, the Benjamini, Krieger, and Yekutieli procedure, based on the Benjamini-Hochberg method, was applied to both the pulmonary function and cytokine datasets. Adjusted *q* values < 0.05 were considered statistically significant.

In the TLR4 inhibition group, the transplanted lungs appeared visibly clear, and the wet-to-dry weight ratio was significantly reduced compared to the control group (Figure 1B, C). Regarding transplanted lung function, the TLR4 inhibition group demonstrated significantly lower mean airway pressure (Figure 1D), while compliance and inspiratory volume were significantly higher (Figures 1E,F). ELISA analysis of transplanted lung lysates revealed that MCP-1, a key mediator of inflammatory macrophage aggregation,^8^ was significantly reduced in the TLR4 inhibition group (Figure 2A). Additionally, inflammatory cytokines involved in the recruitment of inflammatory cells or released by them, including IFN-γ and IL-6, were significantly lower in the TLR4-treated group compared to the control group (Figures 2B and C). TGF-β, one of the central cytokines driving the fibrotic process by inducing fibroblast proliferation and collagen deposition,^9^ tended to be lower in the TLR4 inhibition group. (Figure 2D) Representative H&E-stained histological images of the lung tissues are presented in Figure 3A. The histological scores of acute lung injury were significantly lower in the TLR4 inhibition group than in the control group (Figure 3B). Representative immunohistochemical staining images of lung tissue are presented in Figure 3C. The number of infiltrating iNOS-positive inflammatory cells, including pro-inflammatory M1 macrophages, monocytes, and neutrophils, known to be stained by iNOS,^10^ was significantly reduced in the TLR4 inhibition group. Additionally, CD206-positive cells, which contribute to tissue repair and wound healing as M2 macrophages,^11^ showed a tendency to decrease in the TLR4 inhibition group (Figure 3D).

**Figure 2:**
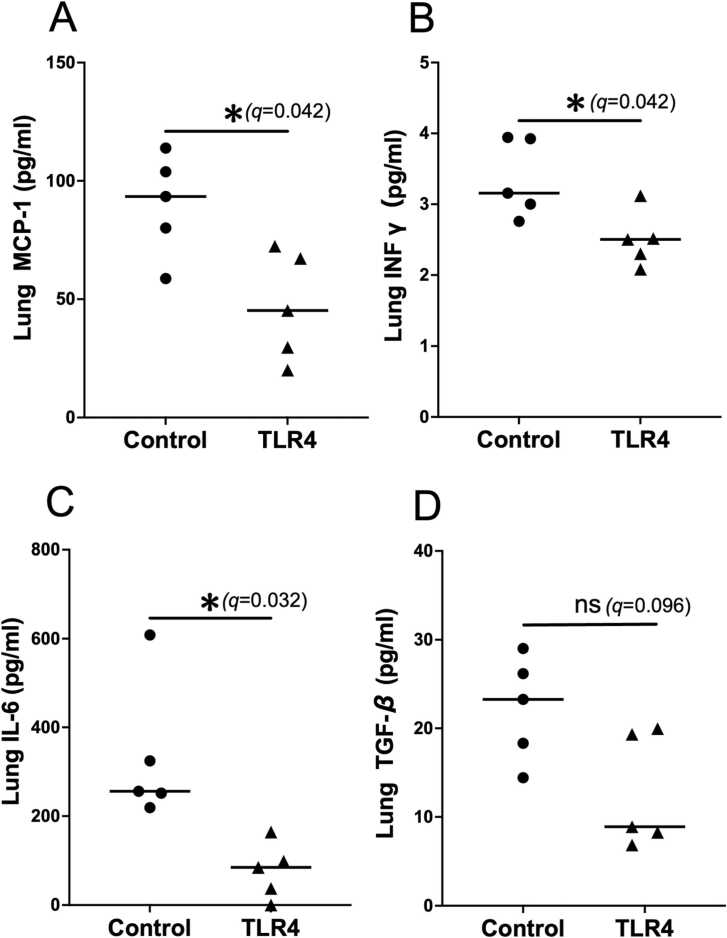
*TLR4 inhibition significantly suppressed inflammation in the allograft.***(A-C)** Enzyme-linked immunosorbent assay (ELISA) analysis of MCP-1 **(A)**, IFN-γ **(B)**, IL-6 **(C)**, and TGF-β **(D)** in lysates from transplanted lung. Sample size: *n* = 5 for each group. *q < 0.05, ns: not significant. Bar indicates the mean values.

**Figure 3:**
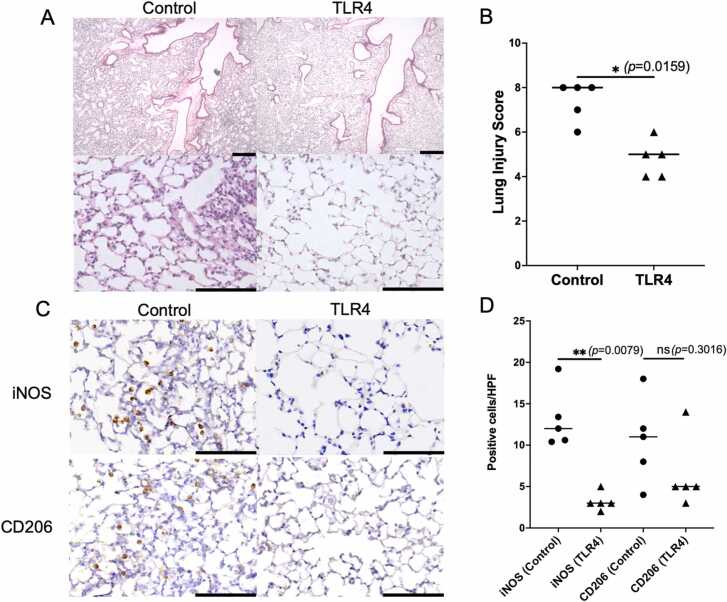
*iNOS-positive inflammatory cells and CD206-positive M2 macrophages were reduced in the allograft, with a more pronounced reduction observed in inflammatory cells*. **(A)** Hematoxylin and eosin staining. Scale bar: 100 µm. **(B)** Comparison of lung injury score. **(C)** Representative immunohistochemistry images showing iNOS and CD206 staining in longitudinal sections of allografts. Scale bar: 100 µm. **(D)** Quantitative analysis of iNOS- and CD206-positive staining in the allograft. Sample size: *n* = 5 for each group. *P < 0.05, **P < 0.01, ns: not significant. Bar indicates the mean values.

In our study, we demonstrated that the inhibitory peptide targeting TLR4 significantly reduced inflammatory cell infiltration, evidenced by decreased numbers of iNOS-positive cells, and lowered levels of pro-inflammatory and fibrogenetic cytokines such as MCP-1, IFN-γ, IL-6, and TGF-β within the lung allograft. This attenuation of inflammation was associated with reduced vascular permeability, as reflected by a lower wet-to-dry weight ratio, ultimately resulting in improved lung function during the IRI phase following lung transplantation.

Limitations of this study include the fact that, although we showed that TLR4 inhibition effectively attenuates inflammation associated with IRI, our experimental design does not allow us to distinguish whether these protective effects originate from inhibition in the donor lung, the recipient immune system, or both. In addition, we did not include a sham group to provide baseline values reflecting the impact of surgery itself; its inclusion would have enabled a more accurate evaluation. Furthermore, we did not utilize TLR4- or MyD88-knockout mouse models, which could have provided further mechanistic insights into the specific pathways involved. Finally, while TLR4 inhibitors have demonstrated efficacy in murine models of sepsis, prior human clinical trials using a pharmacological antagonist of the MD2–TLR4 complex in patients with severe sepsis failed to show a significant difference in the primary endpoint of 28-day all-cause mortality, despite comparable side effect profiles between treatment and placebo groups.^12^ This highlights the challenge of translating promising preclinical findings into clinical benefit.

Our findings demonstrate that VIPER, an 11-amino-acid viral inhibitory peptide that blocks TLR4 signaling, exerts a therapeutic effect in a clinically relevant murine lung transplantation model. This supports the potential of TLR4-targeting therapies to improve transplant outcomes by mitigating IRI-associated inflammation.

In conclusion, our results indicate that the inhibitory peptide targeting TLR4 primarily modulates the inflammatory response during IRI, a key driver of graft dysfunction. By reducing inflammatory and fibrogenetic cytokine production and the recruitment of immune cells into the allograft, TLR4 inhibition contributes to improved lung function in the early post-transplant period. These findings support further investigation of TLR4-targeting strategies as potential therapeutic interventions in human lung transplantation.

## CRediT authorship contribution statement

YG, IY, YS, ND, and MT conducted the experiments. YG and MT drafted the manuscript. ST, AO, DN, and HD contributed to the study design and reviewed and revised the manuscript. YG and TM analyzed and interpreted the data and contributed to manuscript writing. HK reviewed and analyzed the pathological findings. MT and HD designed and supervised the study. All authors have read and approved the final version of the manuscript.

## Financial support

MT was supported by JSPS KAKENHI (23K19632).

## Disclosure statement

The authors declare that they have no known competing financial interests or personal relationships that could have appeared to influence the work reported in this paper. The authors of this manuscript have no conflicts of interest to disclose as described by JHLT Open*.*
